# Factors Associated With Weight Management Referral Acceptance and Uptake in Kidney Transplant Candidates

**DOI:** 10.1111/ctr.70659

**Published:** 2026-08-28

**Authors:** Linda‐Marie Lavenburg, Harry Morford, Susan Stark, Sanjana Kamat, Mariela Sierra Mendoza, Michele Molinari, Chethan Puttarajappa

**Affiliations:** ^1^ Department of Medicine University of Pittsburgh Pittsburgh Pennsylvania USA; ^2^ Starzl Transplant Institute University of Pittsburgh Medical Center Pittsburgh Pennsylvania USA; ^3^ Conemaugh Health System Conemaugh Memorial Medical Center Johnstown Pennsylvania USA

**Keywords:** kidney disease, obesity, outpatient care

## Abstract

**Introduction:**

Obesity is a significant barrier to kidney transplantation (KT). KT candidates with obesity are often referred to weight management programs (WMP) but patients' acceptance for WMP referral and their subsequent follow‐through are unknown.

**Design and Methods:**

Using a single‐center study, we evaluated the effectiveness of referrals to medical or surgical WMPs among KT candidates to initiate obesity care. We examined factors associated with patients establishing care and any effects of referral acceptance on BMI, KT waitlisting and transplantation.

**Results:**

The median age was 57 years, 49% were male, 56% were White, 79% were on dialysis, and 53% were from high social disadvantage areas. Thirty patients (50.8%) accepted WMP referral but only 9 (15.3%) contacted WMP. Patients accepting referral had higher BMI [median 47 vs. 45; *p* = 0.002] and resided closer to the KT center [20 vs. 48 miles; *p* = 0.010]. In multivariable models, referral acceptance decreased 2% with each mile away from KT center (OR 0.98; 95%CI: 0.96–1.00) and increased by 22% with every 1 unit increase in BMI (OR 1.22, 95%CI: 1.03–1.45). Among patients accepting referral, every 1‐year increase in age reduced odds of contacting WMP by 17% (OR 0.83, 95%CI: 0.74–0.96).

**Conclusion:**

A large share of KT candidates with obesity do not establish care with WMPs. Increasing age and distance to KT center were contributing factors to low referral acceptance and engagement. Efforts aimed at obesity management in KT should consider these barriers. WMPs integrated within KT programs may overcome barriers to establishing care with WMPs.

## 1 Introduction

For patients with end‐stage kidney disease (ESKD), kidney transplantation (KT) confers better long‐term survival benefits [1, 2], and quality of life [3]. However, access to KT is influenced by multiple factors, including demographics, comorbidities, and psychosocial determinants. Of these, obesity, defined by a body mass index (BMI) ≥ 30 kg/m^2^, affects over 420 000 people with ESKD [4]. Despite KT benefit to reduce mortality risk by 66% for individuals with BMI ≥ 35 kg/m^2^, and 48% risk reduction for those with BMI ≥ 40 kg/m^2^ (Class 3 obesity) [5], obesity remains a relative contraindication to KT across many transplant centers [6, 7, 8, 9].

BMI requirements for KT vary across centers, but most require a BMI < 35–40 kg/m^2^ [6, 7, 8, 10]. Patients who exceed these thresholds are often instructed to lose weight without structured support, and most never achieve eligibility [6, 11]. Although national kidney guidelines recommend multidisciplinary weight‐loss strategies—including dietary counseling, pharmacotherapy, and bariatric surgery—for candidates with BMI ≥ 35 kg/m^2^ [12], obesity management in kidney disease remains limited. National kidney organizations endorse integrated approaches [13], yet only 19% of United States KT programs report having an embedded weight management programs (WMPs) [14, 15, 16, 17].

Highly effective obesity‐modifying medications have renewed optimism around medical weight loss [12, 14, 15, 18], but access remains uneven. Patients with advanced kidney disease commonly face barriers to engaging in WMPs or initiating newer therapies, including transportation challenges, cost and insurance restrictions, medication side effects, and the time burden of dialysis [16, 17]. Despite these obstacles, little is known about how KT candidates actually use available WMP resources. Therefore, we sought to examine WMP utilization and factors associated with engagement among KT candidates with class 3 obesity.

## 2 Design and Methods:

### 2.1 Study Design

Prospective quality improvement (QI) initiative approved by University of Pittsburgh Medical Center (UPMC) and conducted from 7/1/2022–6/30/2024. Because electronic records did not capture acceptance or decline of WMP referrals, this QI initiative was developed to track referral uptake and standardize obesity education and counseling for KT candidates. The initial phase concluded after two years following the identification of the need to revise the KT‐to‐WMP referral workflow.

### 2.2 Study Population

The study cohort consisted of individuals with ESKD, a BMI ≥ 40, and referred for KT evaluation at UPMC between July 2022 and June 2024.

### 2.3 Counseling and Intervention

All patients referred for transplantation with a BMI ≥ 40 kg/m^2^ were offered weight counseling and a referral to a WMP. In conjunction with BMI, comorbidities, functional status, and other clinical factors were considered in the final candidacy decision; however, they did not influence eligibility for weight counseling or WMP referral. Individuals with BMI 40–43.9 kg/m^2^ were eligible for KT evaluation and encouraged to optimize weight toward a BMI closer to 35 kg/m^2^ through monthly telephone counseling with a Registered Dietitian (RD). (Figure S1).

Individuals with BMI ≥ 44 kg/m^2^ were required to reduce BMI < 44 kg/m^2^ before KT evaluation. They were informed by phone of weight‐related ineligibility and offered WMP referral to a comprehensive medical weight‐loss program and/or bariatric surgery, offering telemedicine or in‐person visits, but not integrated with the transplant program. Selection of WMP referral was based on personal preference. KT staff did not provide weight management guidance based on clinical factors, weight loss potential, and insurance coverage when referrals were offered. Those declining referrals were offered opt‐in monthly RD counseling.

Standardized opt‐in RD counseling consisted of a 30 min telephone session focused on meal planning, portion control, and low‐calorie eating strategies. The RD did not counsel on selection of medical weight management or bariatric and metabolic surgery. Individuals unreachable for two consecutive months were considered lost to follow‐up.

### 2.4 Data Collection

We collected demographic and clinical data to assess their impact on WMP referral acceptance. We also collected social determinants of health factors, including employment status, zip‐codes, and miles to the KT center (UPMC). We used the zip‐codes to obtain the Area Deprivation index (ADI), a validated measure of income, education, employment, and housing quality [19, 20]. ADI national scores range 1–100, with higher scores indicating greater disadvantage [20, 21]. ADI scores were categorized as <80 (low) or ≥ 80 (high), since scores ≥ 80 are associated with poor health outcomes [19].

Patients were followed prospectively after initial contact by the RD, and follow‐up data on engagement with the WMP, changes in BMI, waitlist status, and transplant status were collected through in‐depth chart reviews.

### 2.5 Outcomes

Primary outcome was acceptance of WMP referral. We evaluated for individual‐level factors associated with referral acceptance and subsequent contact with WMP. Secondary outcomes were change in BMI over 12 months and proportion who achieved KT waitlist status.

### 2.6 Covariates

We examined whether patients accepted WMP referrals based on their age, gender, race, BMI, dialysis status, miles from the transplant center, employment, insurance, and ADI.

### 2.7 Statistical Analysis

Differences in baseline characteristics between those who accepted or declined referral to WMP, and between those who did and did not contact a WMP were evaluated with Student's *t* test, Wilcoxon rank‐sum test or Kruskal–Wallis test for continuous variables, and chi‐square test for categorical variables. Results are presented as mean (or medians) and proportions. We used unadjusted and adjusted multivariable logistic regression models to determine the odds of referral acceptance. Covariates with *p*‐value < 0.1 on univariable analysis were included in the multivariable model. Missing data was highest for employment status (29%). We performed a complete case analysis. Differences between baseline and 2‐year BMI were evaluated using the Wilcoxon rank‐sum test. Exploratory analyses of between‐group differences in BMI trajectories over time were performed using linear mixed‐effects models with repeated BMI measurements at baseline, 1 year, and 2 years. Analysis was performed with Stata version 17 (StataCorp, College Station, TX). A two‐sided *p*‐value of <0.05 was considered statistically significant.

## 3 Results

**FIGURE 1:**
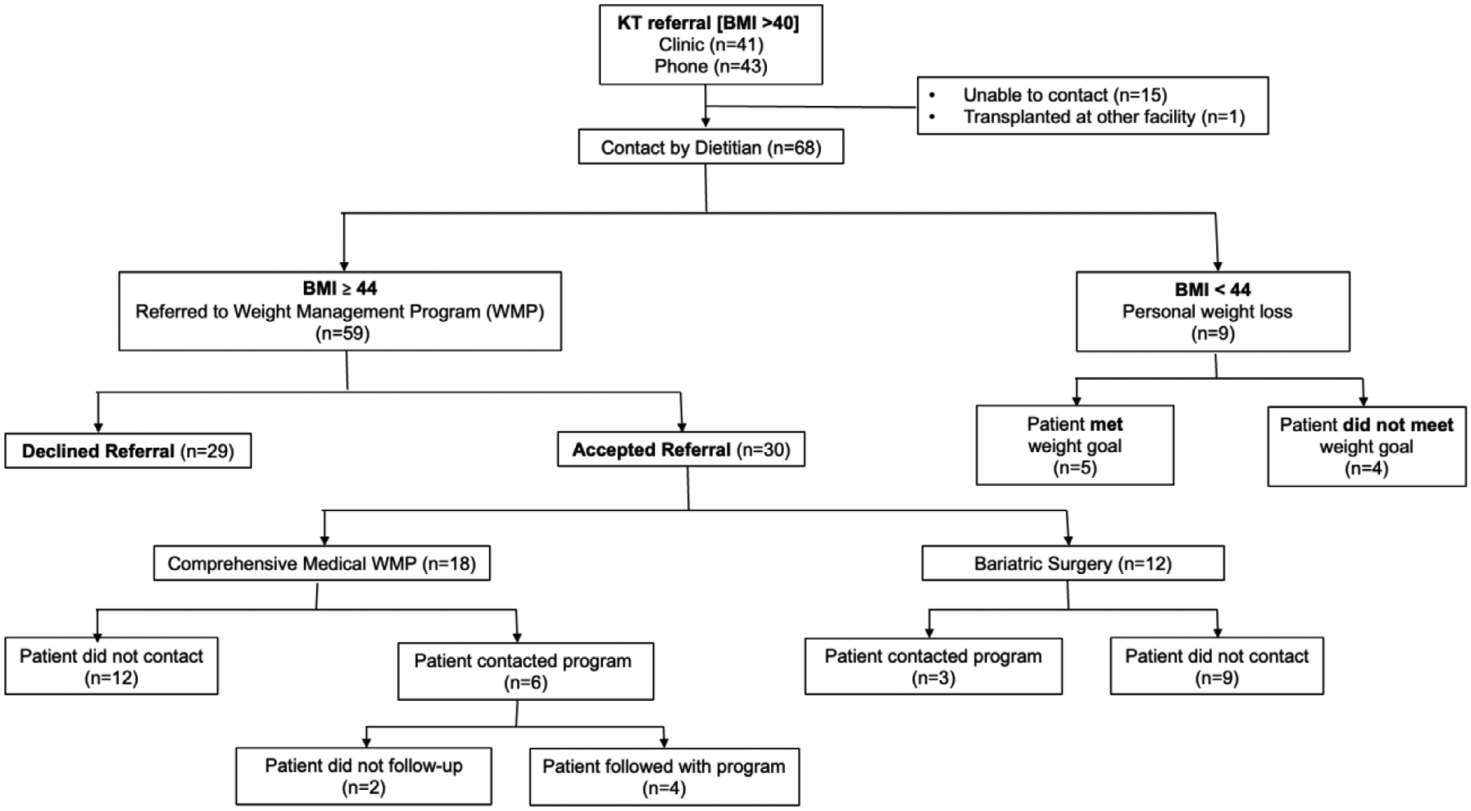
Cohort diagram.

Between 2022–2024, 83 individuals with a BMI ≥ 40 kg/m^2^ were referred for KT evaluation. Fifteen could not be contacted or underwent KT evaluation elsewhere (Figure 1). The final cohort included 68 individuals contacted by the RD, of whom 59 (87%) with a BMI ≥ 44 were offered a WMP referral. Median age was 54 years (IQR 46–65), 47% were male, 53% White, 47% were Black or from other racial/ethnic group. Most had ESKD with 79% receiving renal replacement therapy and 53% had ADI ≥ 80. (Table 1)

**TABLE 1:** Differences in characteristics of patients who accepted or declined weight loss program referrals. Observations for differences in BMI and weight after 1 year were non‐significant, further analysis needed.

Variable	All (*N* = 59)	Declined referral (*N* = 29)	Accepted referral (*N* = 30)	*p* value
Age, median (IQR)	57 (49–65)	61 (52–68)	55 (49–64)	0.27
Male, n (%)	29 (49%)	17 (59%)	12 (40%)	0.15
Race, n (%)				0.73
White	32 (56%)	17 (59%)	15 (53%)	
Black	22 (39%)	10 (34%)	12 (43%)	
Other	3 (5%)	2 (7%)	1 (4%)	
BMI, kg/m^2^, median (IQR)	46 (44–50)	45 (41–46)	47 (45–50)	**< 0.01**
Employment Status, *n* (%)				0.58
Working (full or part time)	16 (38%)	6 (33%)	10 (42%)	
Not working	26 (62%)	12 (67%)	14 (58%)	
Dialysis, *n* (%)				0.88
Not on Dialysis	10 (21%)	5 (20%)	6 (33%)	
On Dialysis	38 (79%)	20 (80%)	18 (78%)	
Miles from KT center, median (IQR)	37 (4–59)	48 (17–67)	20 (3–41)	**0.01**
ADI, median (IQR)	80 (61–91)	78 (59–87)	87 (63–94)	0.27
ADI relative to 80, *n* (%)				0.52
< 80	28 (47%)	15 (52%)	13 (43%)	
≥ 80	31 (53%)	14 (48%)	17 (57%)	
Prior KT, *n* (%)	2 (3%)	2 (7%)	0 (0%)	0.14
Insurance, *n* (%)				0.44
Commercial	44 (77%)	24 (83%)	20 (71%)	
Medicare	9 (16%)	3 (10%)	6 (21%)	
Medicaid	1 (2%)	1 (3%)	0 (0%)	
Uninsured	3 (5%)	1 (3%)	2 (7%)	

*Note:* Missingness was highest for employment status (29%) and dialysis history (19%), while race, insurance, and area deprivation index each had 3% missing.

Abbreviations: ADI, area deprivation index; BMI, body mass index; IQR, interquartile range; KT, kidney transplantation.

Of those offered a referral, 30 patients (51%) accepted referral. Of the 9 individual‐level covariates analyzed, age, BMI and distance were significantly associated with referral acceptance. Patients accepting referral had a higher median BMI [47 (IQR 45–50) vs. 45 (IQR 41–46); *p* = 0.002] and resided closer to the KT center [20 (IQR 3–41) vs. 48 (IQR 17–67) miles; *p* = 0.01]. In multivariable models, referral acceptance decreased 2% with each mile away from KT center (OR 0.98; 95%CI: 0.96–1.00) and increased by 22% (OR 1.22, 95%CI: 1.03–1.45) with every 1 kg/m^2^ increase in BMI. There was no association between ADI and WMP referral acceptance.

Nine (30%) individuals who accepted referral also contacted a WMP. Those who contacted were younger than those who did not contact WMP (median age 42 [IQR 38–54] vs. 59 [IQR 53–65]; *p* = 0.004), but other characteristics were similar (Table S1). Among patients accepting the referral, every 1‐year increase in age reduced odds of contacting WMP by 17% (OR 0.83, 95%CI: 0.74–0.96).

BMI data at 1‐year follow‐up were available for 26 of 30 who accepted referrals and 24 of 29 who declined. Median BMI change was −0.93 kg/m^2^ (IQR −2.78‐0) among those accepting referral and −0.45 kg/m^2^ (IQR −1.67‐0) among those declining referral (*p* = 0.69). At 1 year, 6 (25%) individuals who declined and 1(3.9%) who accepted the WMP referral achieved a BMI < 40 kg/m^2^ (*p* = 0.03).

BMI data at 2‐year follow‐up were available for 22 of 30 who accepted referral and 19 of 29 who declined. Mean BMI change from baseline to 2‐years was −2.96 (SD 4.36) among those accepting referral compared to −3.81 (SD 4.88) among those declining referral, with no significant between‐group difference.

Of 59 patients who were offered WMP, at 1‐year follow‐up, 32 (54.2%) had not completed a KT clinic evaluation, 23 (39.0%) were seen but declined a transplant, and only 4 (6.8%) had been waitlisted. At 2‐year follow‐up, all who accepted WMP referral were not waitlisted or transplanted and no longer followed by KT. Of the 29 who declined referral, 3 (10.3%) were waitlisted, and 1 (3.5%) was transplanted; the remaining were removed from the KT evaluation process.

## 4 Discussion

Prior studies have reported obesity treatment utilization as low as 7% among qualifying individuals in primary care, but similar data in KT candidates with obesity is not well defined [22]. In a single large academic health system, we offered WMP referrals to individuals interested in KT but ineligible due to Class 3 obesity. Despite compelling need to reduce weight for KT waitlist eligibility, only half of those offered a WMP referral accepted it, and 1/3 of people who accepted a referral contacted the WMP. This amounts to a palpable care gap, especially since patients with BMI ≥ 35 have lower rates of KT waitlisting [4] and have greater mortality risk on dialysis compared to transplant recipients [5].

Reasons for declining medical or surgical weight‐management referral among people with ESKD are unclear but likely multifactorial. Saeed et al. found that most preferred diet and exercise, and fewer than 40% preferred education [23]. Bariatric surgery and weight‐loss medications were least preferred, which may explain low referral acceptance in our cohort. We also found that willingness to join a WMP increased with BMI. Patients near BMI eligibility thresholds may believe they can reach BMI goals on their own. More people who declined referral achieved BMI goals than those who accepted referral, but larger studies are needed to determine whether BMI influences this relationship. Importantly, most participants in our cohort did not reach their target BMI, underscoring the need for multi‐level interventions to help overcome barriers to successful weight management.

Higher miles from the KT center were a barrier to accepting WMP referrals. Transplant institutes often evaluate prospective candidates from a large radius. Longer travel distances may reduce engagement with obesity management programs due to financial, travel, insurance, and time‐related barriers, as well as preference for receiving care closer to home. The expansion of telehealth technology during the COVID‐19 pandemic offers potential solutions to address distance‐related barriers. Additional strategies to improve engagement may include integrating WMP within transplant programs, improving communication with primary care providers and nephrologists to reinforce BMI goals, and facilitating access to obesity care closer to their homes.

Similar interventions may address advancing age as a barrier to care. Older adults with advanced kidney disease have high comorbidity burden, fatigue [24], and frailty [25]. These may discourage participation in physical activity, meal preparation and/or transportation to medical appointments [25, 26] despite recognition of their potential benefits.

To improve the KT‐to‐WMP referral workflow, we created an integrated weight management–nephrology clinic that received direct referral notifications and contacted patients to schedule visits with a dual‐trained obesity medicine and nephrology specialist. Over 8 months, 63 patients with advanced kidney disease and Class 3 obesity were offered referral; 43 accepted, and 26 (61%) completed or scheduled an initial visit, while 17 (39%) awaited scheduling. This model doubled WMP engagement in less than half the time of our prior two‐year initiative. Contributing factors likely included increased trust in a dual‐trained specialist and the simplified, patient‐centered referral pathway. Continued follow‐up is needed to assess its impact on KT waitlisting; however, emerging evidence supports the effectiveness of such integrated WMPs in KT [27, 28].

Our study is among the few to quantify the utilization of WMP by individuals with advanced kidney disease seeking KT and presents two years of data from a large academic tertiary care center. Limitations include small sample size, inability to determine use of outside WMPs, single‐center design, and lack of direct measurement of patient‐, provider‐, and system‐level barriers to referral acceptance and uptake. Between‐group differences in BMI trajectories and transplant outcomes were not significant, and should be interpreted cautiously, as this QI initiative was not powered to evaluate these outcomes. Additional unmeasured factors likely contributed to low WMP engagement, including appointment availability, transportation barriers, competing medical priorities, and insurance restrictions. Because reasons for referral decline and non‐engagement were not systematically collected, this study could not distinguish logistic barriers from patient preference or readiness for treatment. The lack of detailed process metrics reflects a common limitation of real‐world QI workflows, where operational feasibility may precede comprehensive mechanistic assessment. Additionally, we did not collect data on the use of obesity medications such as glucagon‐like‐peptide‐1 receptor agonists and other incretin mimetics, since the study occurred during a period when the role and safety of these obesity treatments in people with kidney disease were evolving. Future work should incorporate qualitative interviews or structured surveys to better characterize barriers to obesity treatment among KT candidates and re‐evaluation of weight management referral acceptance and uptake in the era of novel incretin mimetics.

In conclusion, our findings from a center with extensive obesity treatment resources underscore substantial underutilization of obesity treatment among KT candidates. Distance from the KT center and older age were associated with low referral acceptance and engagement. However, they likely represent only a limited component of the broader structural, socioeconomic, and individual‐level barriers, since patient‐, provider‐, and system‐level barriers were not evaluated. Early efforts to streamline referrals and embed a WMP within the KT clinic are promising, but longer follow‐up is needed to assess effects on KT waitlisting and transplantation.

## Author Contributions

Concept/design: Susan Stark, Linda Marie‐Lavenburg, Michele Molinari, Chethan Puttarajappa. Data analysis/interpretation: Linda Marie‐Lavenburg, Harry Morford, Sanjana Kamat, Mariela Sierra Mendoza, Michele Molinari, Susan Stark, Chethan Puttarajappa. Drafting article: Linda Marie‐Lavenburg, Harry Morford, Chethan Puttarajappa. Critical revision of article: Linda Marie‐Lavenburg, Harry Morford, Sanjana Kamat, Mariela Sierra Mendoza, Michele Molinari, Susan Stark, Chethan Puttarajappa. Approval of article: Linda Marie‐Lavenburg, Harry Morford, Sanjana Kamat, Mariela Sierra Mendoza, Michele Molinari, Susan Stark, Chethan Puttarajappa. Statistics: Harry Morford, Chethan Puttarajappa. Data collection: Harry Morford, Mariela Sierra Mendoza, Susan Stark, Chethan Puttarajappa Linda‐Marie Lavenburg

## Funding

Dr. Lavenburg is supported by American Heart Association Career Development Award (https://doi.org/10.58275/AHA.24CDA1273478.pc.gr.193549).

## Conflicts of Interest

Linda‐Marie Lavenburg participates in investigator‐initiated research sponsored by Pfizer and has received a career development award from Robert A. Winn/Bristol Myers Squibb Foundation. Chethan Puttarajappa is a consultant for Replimune Inc., unrelated to the submitted work.

## Supporting information




**Supporting File 1:** ctr70659‐sup‐0001‐SuppMat.docx

## Data Availability

The data that support the findings of this study are available on request from the corresponding author. The data are not publicly available due to privacy or ethical restrictions.
