# Supplementary material for: Factors Associated With Weight Management Referral Acceptance and Uptake in Kidney Transplant Candidates
Source: Clin Transplant. 2026 Aug 28;40(9):e70659. doi: 10.1111/ctr.70659 (PMC13525081; doi:10.1111/ctr.70659)
Supplement: Supplementary file 1 [file CTR-40-e70659-s001.docx]

**Supplemental material**

**Supplement Table 1.** Differences in characteristics of patients who did or did not contact weight loss program

**Supplement Figure 1**. Weight management referral workflow

**Supplement Table 1:** Differences in characteristics of patients who did or did not contact weight loss program

| **Variable** | **All**  **(N=30)** | **Did not contact WMP**  **(N=21)** | **Contacted WMP (N=9)** | **p value** |
| --- | --- | --- | --- | --- |
| Age, median (IQR) | 55 (49-64) | 59 (53-65) | 42 (38-54) | **< 0.01** |
| Male, n (%) | 12 (40) | 9 (43) | 3 (33) | 0.63 |
| Race, n (%) |  |  |  | 0.21 |
| White | 15 (54) | 12 (60) | 3 (38) |  |
| Black | 12 (43) | 8 (40) | 4 (50) |  |
| Other | 1 (4) | 0 (0) | 1 (13) |  |
| BMI, kg/m^2^, median (IQR) | 47 (45-50) | 46 (45-5) | 49 (47-51) | 0.14 |
| Employment Status, n (%) |  |  |  | 0.94 |
| Working (full or part time) | 10 (42) | 7 (41) | 3 (43) |  |
| Not working | 14 (58) | 10 (59) | 4 (57) |  |
| Dialysis, n (%) |  |  |  | 0.50 |
| Not on Dialysis | 5 (20) | 2 (29) | 3 (17) |  |
| On Dialysis | 20 (80) | 5 (71) | 15 (83) |  |
| Miles from KT center, median (IQR) | 20 (3-41) | 34 (5-46) | 7 (3-37) | 0.40 |
| ADI, median (IQR) | 87 (63-94) | 90 (64-94) | 80 (38-92) | 0.46 |
| ADI relative to 80, n (%) |  |  |  | 0.94 |
| <80 | 13 (43) | 9 (43) | 4 (44) |  |
| ≥80 | 17 (57) | 12 (57) | 5 (56) |  |
| Insurance, n (%) |  |  |  | 0.44 |
| Commercial | 24 (83) | 7 (78) | 17 (85) |  |
| Medicare | 3 (10) | 2 (22) | 1 (5) |  |
| Medicaid | 1 (3) | 0 (0) | 1 (5) |  |
| Uninsured | 1 (3) | 0 (0) | 1 (5) |  |
| **Abbreviations:** IQR-interquartile range; BMI- body mass index; KT- kidney transplantation; ADI- area deprivation index  Missingness was highest for employment status (20%) and dialysis history (17%), with smaller amounts for race (7%), distance from transplant center, and insurance (3%) | | | | |

**Supplement Figure 1:** Weight management referral workflow

Abbreviations: KT- kidney transplantation; BMI- body mass index; RD- registered dietitian; WMP- weight management program
